# *Lactobacillus rhamnosus* Strains Relieve Loperamide-Induced Constipation via Different Pathways Independent of Short-Chain Fatty Acids

**DOI:** 10.3389/fcimb.2020.00423

**Published:** 2020-08-19

**Authors:** Gang Wang, Shurong Yang, Shanshan Sun, Qian Si, Linlin Wang, Qiuxiang Zhang, Gaojue Wu, Jianxin Zhao, Hao Zhang, Wei Chen

**Affiliations:** ^1^State Key Laboratory of Food Science and Technology, Jiangnan University, Wuxi, China; ^2^School of Food Science and Technology, Jiangnan University, Wuxi, China; ^3^Department of Gastroenterology, The Affiliated Wuxi Second People's Hospital of Nanjing Medical University, Wuxi, China; ^4^International Joint Research Laboratory for Probiotics, Jiangnan University, Wuxi, China; ^5^(Yangzhou) Institute of Food Biotechnology, Jiangnan University, Yangzhou, China; ^6^National Engineering Research Center for Functional Food, Jiangnan University, Wuxi, China; ^7^Wuxi Translational Medicine Research Center and Jiangsu Translational Medicine Research Institute Wuxi Branch, Wuxi, China; ^8^Beijing Innovation Centre of Food Nutrition and Human Health, Beijing Technology and Business University, Beijing, China

**Keywords:** slow transit constipation, *Lactobacillus rhamnosus*, serotonin, neurotrophic factor, short-chain fatty acids, gut microbiota

## Abstract

Increasing researches have confirmed the relationship between slow-transit constipation and gut microbiota dysbiosis. Many population and animal experiments have identified probiotics as effectors for the relief of constipation symptoms, but the specific mechanism remains unclear. In this intervention study, *Lactobacillus rhamnosus* strains isolated from five different sources were administered to mice with loperamide-induced constipation, and the impacts of these strains on constipation-related indicators were evaluated. All five strains of *L. rhamnosus* were found to improve constipation to various degrees. However, contrary to previous studies, the abilities of *L. rhamnosus* strains to improve constipation symptoms were not associated with the levels of short-chain fatty acids (SCFAs) in the colon. The effects of different strains of *L. rhamnosus* on constipation relief were associated with different aspects of the GI tract, including gastrointestinal regulatory peptides, neurotransmitters, neurotrophic factors, and gut microbiota. The findings of this study demonstrate that *L. rhamnosus* strains can alleviate constipation-related symptoms via different pathways independent of SCFAs regulation. This study yields a new perspective for clinical use of probiotics to better improve constipation symptoms, by combining strains with different mechanisms for alleviation of constipation.

## Introduction

Slow-transit constipation, a common clinical symptom of digestive system dysfunction, is characterized by persistent, infrequent, or incomplete defecation. Accelerated pace of contemporary lifestyle is associated with changes in dietary habit, stress level, and an increased probability of constipation symptoms. About 12–30% of people suffered from constipation in (Choopani et al., 2017). Other studies have shown that the probability of constipation symptoms increases beyond the age of 65 years, and is twice as high in women as in men (Koliani and Lacy, 2017). Constipation not only increases the financial burden of patients, but also increases the social burden of the healthcare system (Sharma and Rao, 2016). Various laxative drugs are being used to alleviate these symptoms. They are rapid in action, but the symptoms of constipation return once drug use has ceased. Additionally, the prolonged use of laxatives can lead to drug dependence and even more severe symptoms of constipation.

The colon is an important “container” for the cultivation of intestinal microbiota. Advances in research have revealed significant roles for these intestinal microbiota in human health. As early as 1998 (Zoppi et al., 1998), studies determined significant differences in gut microbiota between patients with constipation symptoms and their healthy counterparts. Furthermore, the gut microbiota of patients with chronic constipation exhibit characteristic reduction in obligate anaerobic bacteria and corresponding increases in potential pathogenic bacteria and fungi. Also, some studies reported alterations on the abundance of specific species in constipated patients' intestinal microbiota (Zhao and Yu, 2016). Changes in the proportions of these gut microbiota may affect intestinal motility and altering local metabolic environment (Zhao and Yu, 2016). These differences between the gut microbiota of human patients with constipation symptoms and their healthy counterparts suggest that constipation-related symptoms could potentially be relieved via introduction of gut microbiota and/or probiotic which remediate the intestinal microenvironment. A researcher observed a significantly lower abundance of *Bifidobacterium* and *Bacteroides* species in the gut microbiota of patients with functional constipation than in normal controls (Kim et al., 2015). Whereas, after a 2-week course of probiotic VSL#3, the abundance of lactic acid bacteria, *Bifidobacterium* and *Bacteroides* species increased in the normal group but not in patients with constipation. Wang et al. administered *Bifidobacterium adolescentis* to mice with loperamide-induced constipation and found alleviation of symptoms associated with constipation and alteration in gut microbiota (Wang et al., 2017b).

Although considerable evidences indicate that probiotics can alleviate constipation and regulate the gut microbiota, no consistent conclusion has been reached regarding the probiotic mechanisms. The existing research results indicate that probiotics can affect intestinal motility by altering the levels of neurotransmitters and short-chain fatty acids (SCFAs), and by regulating the gut microbiota and immunity (Dimidi et al., 2017). Other study suggested that the intragastric administration of *Bifidobacterium animalis* GCL2505 could effectively relieve the symptoms of loperamide-induced constipation while significantly enhanced the total SCFAs levels in the feces of constipated mice relative to the model group (Aoki et al., 2016). Qian et al. reported that *Lactobacillus fermentum* Lee effectively relieved constipation induced by activated carbon, and up-regulated serum concentrations of acetylcholine and substance P compared with their untreated counterparts with constipation (Qian et al., 2015). What's more, study demonstrated that the daily administration of 4 × 10^10^ CFU of *Lactobacillus reuteri* Shirota improved the frequency of defecation in subjects with constipation and up-regulated the fecal abundance of bifidobacteria (Matsumoto et al., 2010). However, few reports in the literature have described specific differences in the mechanisms by which different species and strains relieve constipation. Therefore, we studied five strains of *Lactobacillus rhamnosus* known to relieve constipation symptoms. We explored the effects of these five strains on the levels of neurotransmitters, SCFAs and gastrointestinal (GI) regulatory peptides, as well as on the intestinal microecology. The differences in these biomarkers among the five strains of *L. rhamnosus* allow clarification of the potential mechanisms by which probiotics alleviate constipation symptoms.

## Materials and Methods

### Preparation of *L. rhamnosus* Suspensions

As shown in Table 1, the strains engaged in this study were obtained from the strain collection of the Food Biotechnology Center at Jiangnan University. To prepare for animal experiments, all strains were cultured in MRS medium and incubated in an anaerobic atmosphere (10% H_2_, 10% CO_2_, and 80% N_2_) in anaerobic chamber (DG250, Don Whitley, UK) for 16 h at 37°C. The strains were subcultured at least three times with 2% (v/v) inoculum. Bacterial cells were collected with centrifuge (6,000 × g, 12 min, 4°C), and then washed with a sterile saline solution twice and stored at −80°C in 30% sucrose solution until use. Before intragastric administration, the bacterial cell suspension was centrifuged and the cells were resuspended in 3% sucrose solution at a density of 5 × 10^9^ CFU/mL.

**Table 1:** Isolates and culture conditions used in this study.

**Species**	**Strain**	**Number**	**Source**	**Culture conditions**
*L. rhamnosus*	CCFM1068	1068	CCFM	37°C, MRS, Anaerobic
*L. rhamnosus*	FFJND15-L2	15-L2	CCFM	37°C, MRS, Anaerobic
*L. rhamnosus*	FHeNJZ7-1	7-1	CCFM	37°C, MRS, Anaerobic
*L. rhamnosus*	FTJDJ11-1	11-1	CCFM	37°C, MRS, Anaerobic
*L. rhamnosus*	FZJHZ11-7	FZJHZ11-7	CCFM	37°C, MRS, Anaerobic

*CCFM, Culture Collection of Food Microorganisms at Jiangnan University*.

### Animals and Experimental Design

Forty-two 6-week-old male SPF grade BALB/c mice were obtained from Shanghai Slack Company. The animal experimental protocol was approved by the Ethics Committee of Jiangnan University (JN. No20180115b1920520) and implemented in accordance with existing EU guidelines (2010/63/EU). The breeding environment was maintained at a temperature of 23 ± 2°C and relative humidity of 50 ± 10%, with a 12-h/12-h light/dark cycle. All animal experiments were performed in strict accordance with the regulations of the Animal Management and Use Committee of Jiangnan University (SYXK 2012-0002).

The mice began to gavage daily after 7 days of adaptation to the environment. During Days 8–25, mice in the normal and model group were administered 0.2 mL of 3% sucrose solution intragastrically daily, while the other five groups were administered 0.2 mL of bacterial suspension (5 × 10^9^ CFU/mL in 3% sucrose solution) daily. Subsequently all the mice except those in the normal group were gavaged with loperamide hydrochloride daily during Days 26–30, while the mice in the normal group was administered distilled water (vehicle) orally daily. The loperamide hydrochloride or distilled water treatment were performed 1 h before bacterial suspension or sucrose solution gavage daily from Days 26–30.

### Determination of Water Content in Feces

On Day 29, after the gavage treatments, the mice were placed in a cage containing absorbent paper. The feces were collected and their wet weight was measured. The fecal matter was lyophilised, and the final weight was recorded as the dry weight. The water content of the feces was calculated according to the following formula:

$$\begin{array}{l} \text{Fecal water content}\left(\%\right)=\frac{\text{wet weight}\left(\text{g}\right)-\text{dry weight}\left(\text{g}\right)}{\text{wet weight}\left(\text{g}\right)}\times 100 \end{array}$$

### Determination of the Time to First Black Stool Defecation

On Day 30, mice in each group were administered activated carbon mixed with *L. rhamnosus* suspensions (or 3% sucrose solution) intragastrically. The mice were then placed into clean, dry cages, and the time when each mouse expelled the first black stool was recorded.

### Determination of the Gastrointestinal Transit Rate

On Day 30, the mice were fasted from food overnight (water was provided). At 8:30 a.m. on Day 31, mice in the normal group were given 0.2 mL of distilled water, while all other mice were given 0.2 mL of 3% loperamide hydrochloride solution (10 mg/kg b.w). After 30 min, all mice were administered activated carbon intragastrically. After 30 min, the mice were sacrificed with pentobarbital sodium (0.5 mL/10 g b.w). The entire small intestine from the pylorus to the caecum was removed, and the total length of the small intestine was measured. The distance to the front edge of the activated carbon was also measured. For each mouse, the GI transport rate was calculated as the percentage of prosthetic advancement of the activated carbon relative to the total length of the small intestine.

### Determination of Peptide, Serotonin, and Neurotrophic Factor Concentrations

Mice that had been fasted for 12 h were injected intraperitoneally a sodium pentobarbital solution (0.5 mL/10 g b.w) and sacrificed. Serum samples were obtained from blood samples by centrifugeation (3,000 × g, 15 min) in which the concentrations of motilin (MTL) and gastrin (GAS) were measured.

The colon tissue was washed with pre-cooled phosphate-buffered saline (PBS). The surrounding adipose tissue was removed, and colon was cut and weighed. The tissue was disrupted using a tissue disrupter in a corresponding volume of PBS (weight to volume ratio of 1:9), and finally centrifuged at 5,500 × g for 10 min. The supernatants were collected and the concentrations of serotonin (5-HT), neurotrophin-3 (NT-3), and brain-derived neurotrophic factor (BDNF) were measured. Refer to the corresponding kit instructions purchased from Shanghai Enzyme Biotechnology Co., Ltd. for specific instructions.

### Fecal SCFA Analysis

For pre-treatment, 20 mg samples of feces were weighed and suspended in 500 μL of saturated NaCl solution to which 20 μL of 10% H_2_SO_4_ was added for acidification. Next, 800 μL of anhydrous ether was added to extract the fatty acids, and the mixture was centrifuged (18,000 × g, 15 min). The upper ether phase was collected and mixed with 0.25 g of anhydrous Na_2_SO_4_. After a 30-min incubation, the mixture was centrifuged at 18,000 × g for 5 min to obtain the upper diethyl ether phase, and the SCFAs in this phase were determined using GC-MS with a Rtx-Wax column (column length: 30 m, inner diameter: 25 μm). The following GC-MS settings were used: carrier gas, He; flow rate, 2 mL/min; injection volume, 1 μL; temperature increase to 140°C at 7.5°C/min, followed by an increase to 200°C at 200°C/min and a 3-min hold and an ionization temperature of 20°C. The whole-scan mode was used for the analysis, and a standard curve was obtained for calculation of each SCFAs' concentration.

### Gut Microbiota Profiling

The mouse fecal samples were subjected to a gut microbiota analysis. A Fast DNA Spin kit (MP Biomedicals, Santa Ana, CA, USA) was used to extract bacterial DNA from the mice fecal samples. The V3–V4 regions of bacterial 16S ribosomal RNA (rRNA) genes were amplified via PCR using barcode-indexed primers (341F: 5′- CCT AYG GGR BGC ASC AG-3′ and 806R: 5′- GGA CTA CNN GGG TAT CTA AT-3′). The PCR products were purified by gel extraction (TIANgel Mini Purification Kit) and then pooled in equimolar concentration. The samples were mixed according to the principle of equal mass concentration, and a library was constructed and sequenced by a MiSeq sequencer (Illumina, San Diego, CA, USA). A TruSeq DNA LT Sample Preparation Kit (Illumina, Santiago, CA, USA) were used to prepare libraries, which were sequenced for 500 + 7 cycles on Illumina MiSeq PE300 platform with a MiSeq Reagent Kit (Illumina, Santiago, CA, USA). Quantitative Insights into Microbial Ecology (QIIME) platform was used for 16S rRNA gene sequencing analysis. Subsequently, pair-end reads with an overlap of >10 bp and without mismatches were assembled. With the trim method of Dada2, the barcode and sequencing primer were trimmed in the assembled sequences. The operational taxonomic unit (OTU) was established de novo with uclust with 97% sequence identity cutoff. The OTUs of Bif-groEL sequences were taxonomically assigned with the Chaperonin Sequence Database and the OTUs of the V3-V4 region were taxonomically assigned with the Ribosomal Database Project (RDP) Naive Bayes classifier. The first sequence in each OTU cluster was selected as the representative sequence, and then aligned to the green genes core set in QIIME with the PyNAST aligner. α-diversity was indicated from the rarefied OTU. β-diversity was evaluated by principal component analysis (PCA) with the center log ratio (clr) transformed genus level count table, along with permutational multivariate analysis of variance (perMANOVA). Microbial biomarkers were differentiated with linear discriminant analysis (LDA) effect size (LEfSe) (logLDA > 2.0 were used as the threshold). The false discovery rate (FDR) control was executed based on the Benjamini-Hochberg procedure to correct for multiple testing. An FDR-adjusted *P*-value < 5% was considered to be significant. For the normalization of data, α-diversity and LEfSe analysis are all analyzed in microbiome analyst (Dhariwal et al., 2017). (https://www.microbiomeanalyst.ca/faces/home.xhtml).

### Statistical Analysis

The statistical analyses were processed with GraphPad Prism 6 and SPSS 21.0. Data are presented as means ± SEM. Because of data sets confirmed with a normal distribution, unpaired Student's *t*-test was used to analyse between the normal group and model group, and one-way ANOVA was used to analyse to compare the effects of model group and *L. rhamnosus* groups, followed by Turkey's *post hoc* test or Dunnett's multiple comparisons test (vs. model group).

## Results

### Significant Differences in the Effect of Individual *L. rhamnosus* Strains on Fecal Water Content

**Figure 1:**
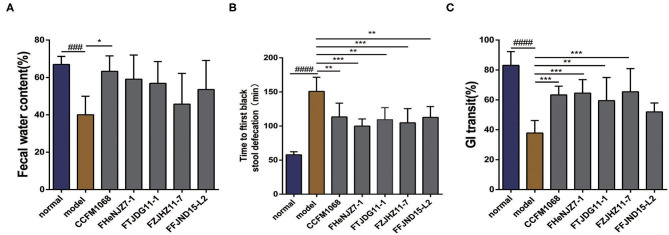
Effects of different strains of *L. rhamnosus* on defecation in a mouse model of constipation. **(A)** Fecal water contents. **(B)** Times to first black stool defecation. **(C)** Gastrointestinal transit rates. Data are means with SEM; Unpaired Student's *t*-test, ^*###*^*P* < 0.001, ^*#####*^*P* < 0.0001 vs. normal group; One-way ANOVA followed by Turkey's *post hoc* test for *L. rhamnosus*-treated groups, *P < 0.05, **P < 0.01, ***P < 0.001 vs. model group for fecal water content. One-way ANOVA followed by Dunnett's multiple comparisons test for *L. rhamnosus*-treated groups, *P < 0.05, **P < 0.01, ***P < 0.001 vs. model group for time to first black stool defecation and gastrointestinal transit rates.

After a 5-day course of loperamide treatment, the water content of feces from mice in the model group is significantly different from that in the normal group (*P* < 0.05), suggesting that treatment reduced the water content and resulted in drier feces. This effect would increase the difficulty of defecation, similar to the clinical symptoms of constipation. As shown in Figure 1A, the water contents in the feces of mice treated with *L. rhamnosus* CCFM1068 differed significantly from that in the model group (*P* < 0.05), suggesting that this strain increased the fecal water content and thus facilitated defecation in mice with loperamide-induced constipation. The notable part of what the result showed is that treatment with other strains of *L. rhamnosus* did not increase the fecal water content in mice with constipation. In other words, *L. rhamnosus* CCFM1068 induced significant differences in fecal water content, indicating strain-related effect.

### No Significant Difference in the Effect of *L. rhamnosus* Strains on the Time to First Black Stool Defecation

The time to the first black stool defecation in mice represents motility throughout the intestine. Specifically, a shorter time to the first black stool defecation indicates more rapid whole intestinal tract motility and a stronger intestinal transport capacity. The results of this experiment are shown in Figure 1B. After 5 days of treatment with loperamide, the time to first black stool defecation in the model group was significantly longer than that in the normal group. In other words, treatment with loperamide resulted in a decrease in the motility and transport capacity of the whole intestine, leading to a prolonged defecation time. However, mice that received an intragastric administration of all the tested *L. rhamnosus* strains exhibited a significantly shorter time to the first black stool defecation than that in the model group (*P* < 0.05). This result indicates that all five *L. rhamnosus* strains can improve the intestinal transit rate significantly in constipation model mice by improving intestinal function and intestinal motility, thus reducing the time to the first black stool defecation. Among these strains, *L. rhamnosus* FHeNJZ7-1 and FZJHZ11-7 showed better capacities in reducing the time of first black stool defecation.

### *L. rhamnosus* Can Significantly Increase the Gastrointestinal Transit Rate in Mice With Constipation

The GI transit rate reflects the dynamics of the small intestine and may thus reflect the effect of *L. rhamnosus* on intestinal motility. A higher GI transit rate (%) indicates a shorter residence of chyme in the small intestine and greater small intestinal motility. The results of this experiment are shown in Figure 1C. After treatment with loperamide, the model group had a significantly lower GI transit rate than the normal group, indicating constipation model was established successfully. After the intragastric administration of *L. rhamnosus* strains except *L. rhamnosus* FFJND15-L2 to mice with constipation exhibited a significantly improved GI transit rate (*P* < 0.05). This observation suggests that treatment with *L. rhamnosus* except for FFJND15-L2 can effectively improve intestinal motility and accelerate the movement and exit of chyme via the small intestine. This together with result in section No significant difference in the effect of *L. rhamnosus* strains on the time to first black stool defecation, indicate that some strains of *L. rhamnosus* might not improve small intestinal motility and colonic motility simultaneously.

### *L. rhamnosus* Strains Showed Diverse Effects on Serum Levels of Gastrointestinal Regulatory Hormones in a Mouse Model of Constipation

**Figure 2:**
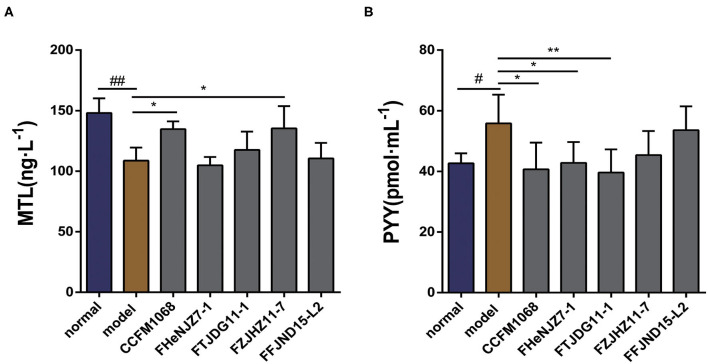
Effects of different *L. rhamnosus* strains on gastrointestinal motility in a mouse model of constipation. **(A)** Motilin (MTL) concentrations in serum. **(B)** Peptide YY (PYY) concentrations in serum. Data are means with SEM; Unpaired Student's *t*-test, ^#^*P* < 0.05, ^*##*^*P* < 0.01 vs. normal group; One-way ANOVA followed by Dunnett's multiple comparisons test for *L. rhamnosus*-treated groups, *P < 0.05, **P < 0.01 vs. model group.

The hormone MTL is a common regulator of GI motility. The serum concentrations of MTL in the mice were evaluated. As shown in Figure 2A, after treatment with loperamide, the serum MTL concentration was significantly lower in constipation model mice than in the normal group, suggesting that the observed reduction in GI transit may be related to the concentration of MTL in the serum. As shown in the figure, the MTL concentrations in the serum of mice treated with *L. rhamnosus* CCFM1068, FZJHZ11-7 were restored to the level in the normal group. These two strains may increase GI transit in mice with constipation by enhancing the serum concentration of MTL. *L. rhamnosus* FHeNJZ7-1 did not enhance the serum concentration of MTL but improved the GI transit rate, suggesting that this strain can enhance intestinal transit via different mechanisms.

As shown in Figure 2B, we observed that after 5 days of loperamide treatment, the serum peptide YY (PYY) concentration was significantly higher in the model group than that in normal group. This observation suggests that loperamide-induced constipation may also be associated with abnormalities in the serum PYY concentration, although the exact mechanism remains unclear. Moreover, we observed that treatment with *L. rhamnosus* CCFM1068, FHeNJZ7-1, and FTJDG11-1 reduced the PYY concentrations in the serum of constipation model mice and alleviated the inhibitory effect of this peptide on GI transit and motility. However, strain FFJND15-L2 and FZJHZ11-7 did not significantly reduce the serum concentration of PYY in constipated mice. This result indicated that *L. rhamnosus* effected changes in the concentration of PYY in serum in a strain-specific manner.

### Differential Effects of *L. rhamnosus* Strains on Intestinal Serotonin in a Mouse Model of Constipation

**Figure 3:**
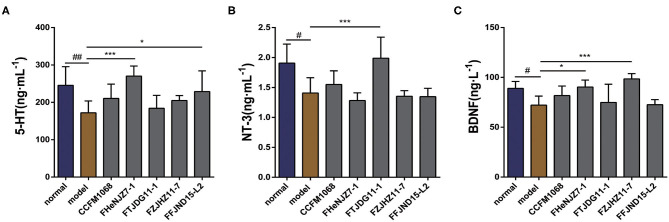
Effects of different strains of *L. rhamnosus* on the concentrations of 5-HT and neurotrophic factors in the colons of constipation model mice. **(A)** 5-HT. **(B)** NT-3. **(C)** BDNF. Data are means with SEM; Unpaired Student's *t*-test, ^#^*P* < 0.05, ^*##*^*P* < 0.01 vs. normal group; One-way ANOVA followed by Dunnett's multiple comparisons test for *L. rhamnosus*-treated groups, *P < 0.05, ***P < 0.001 vs. model group.

Neurotransmitters in the GI tract have important effects on GI motility. As shown in Figure 3A, the administration of loperamide led to a significant downward trend in the level of 5-HT in the colons of constipation model mice. Therefore, constipation symptoms may be related to a decrease in this neurotransmitter in the colon. The administration of *L. rhamnosus* FHeNJZ7-1 and FFJND15-L2 led to significant increases in the concentration of 5-HT in the colon, and the 5-HT concentration of mice in *L. rhamnosus* FHeNJZ7-1 group was significantly higher than that in *L. rhamnosus* FTJDG11-1 group. Taken together, these results indicate that some strains of *L. rhamnosus* can increase the 5-HT concentration specifically in the colon of constipation model mice, thereby enhancing colonic smooth muscle contractions and relieving constipation. However, this effect was clearly and significantly strain-dependent.

### Significant Differences in the Effects of *L. rhamnosus* Strains on Neurotrophic Factors in the Colons of Constipation Model Mice

To explore the potential impacts of *L. rhamnosus* on intestinal nervous system, we first examined NT-3 and BDNF, as shown in Figures 3B,C. Significant decreases in the expression of both NT-3 and BDNF were observed in the tissues of constipation model mice relative to the normal group. This result may be attributed to the activity of loperamide in dysregulation of intestinal nervous system. Only *L. rhamnosus* FTJDG11-1 increased the expression of NT-3 in the colons of constipation model mice. We speculate that this strain effectively relieved constipation by increasing the level of NT-3 in the colon. We further observed significant differences in the abilities of different *L. rhamnosus* strains to increase the expression of NT-3 in the colon.

On the other hand, both *L. rhamnosus* FHeNJZ7-1 and FZJHZ11-7 administration led to significant increases in the expression of BDNF content in the colon when compared to groups treated with the three other bacterial strains and model group. This finding suggests significant differences in the abilities of various *L. rhamnosus* strains to enhance BDNF expression in the colon.

### *L. rhamnosus* Did Not Relieve Constipation by Altering the SCFAs Profile

**Figure 4:**
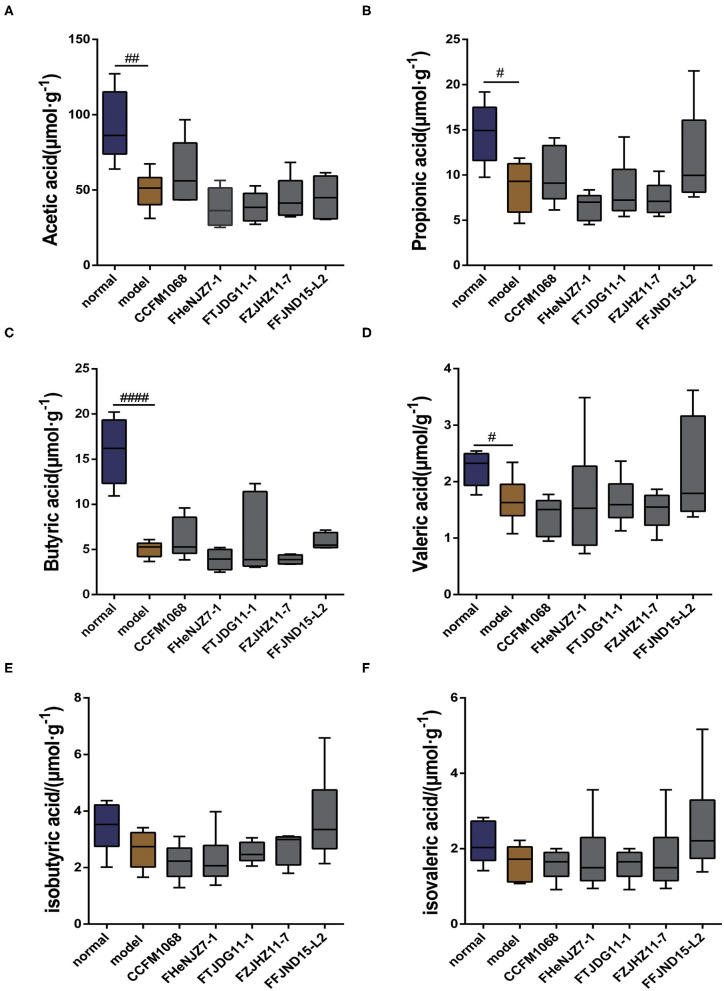
Effects of different strains of *L. rhamnosus* on the short-chain fatty levels in the feces of constipation model mice. **(A)** Acetic acid. **(B)** Propionic acid. **(C)** Butyric acid. **(D)** Valeric acid. **(E)** Isobutyric acid. **(F)** Isovaleric acid. Data are means with SEM; Unpaired Student's *t*-test, ^#^*P* < 0.05, ^*##*^*P* < 0.01, ^*####*^*P* < 0.0001 vs. normal group. One-way ANOVA followed by Dunnett's multiple comparisons test for *L. rhamnosus*-treated groups.

SCFAs are important metabolites produced by intestinal microbiota. Therefore, the SCFAs profile may reflect the effects of *L. rhamnosus* on the gut microenvironment. We evaluated the levels of acetic acid, propionic acid, butyric acid, valeric acid, isobutyric acid, and isovaleric acid in mouse feces. As shown in Figure 4, notably, treatment with loperamide led to significant reductions in the fecal levels of acetic acid, propionic acid, butyric acid, and valeric acid in mice of model group compared to the normal group. However, there were no significant changes in fecal levels of isobutyric acid and isovaleric acid. After administration of *L. rhamnosus*, there were no significant reversal in the concentration of the SCFAs to the levels in the normal group (Figures 4A–D). Since all five strains of *L. rhamnosus* could alleviate constipation, this indicated that the regulation of SCFAs was not necessary the mechanism for these *L. rhamnosus* strains to relieve constipation.

### Differential Effects of *L. rhamnosus* Strains on Gut Microbiota in Constipation Model Mice

**Figure 5:**
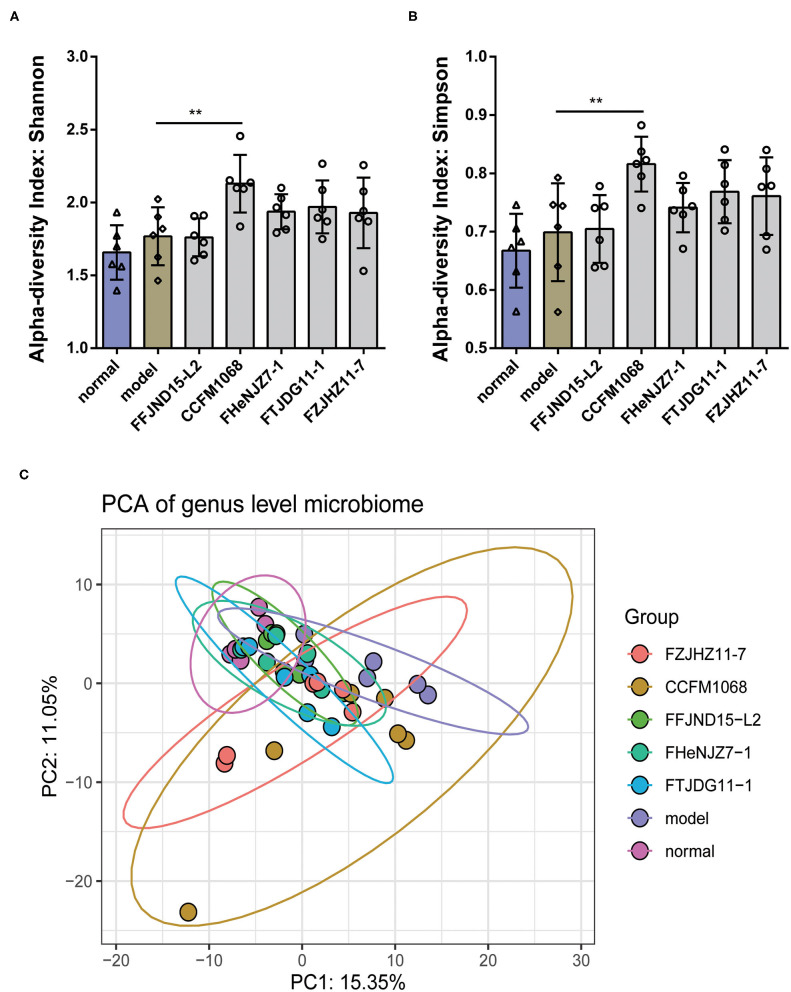
Treatment with *L. rhamnosus* alters the diversity and structure of the gut microbiota. **(A)** α-diversity index: Shannon. **(B)** α-diversity index: Simpson. **(C)** PCA of genus level microbiome. One-way ANOVA for *L. rhamnosus*-treated groups, **P < 0.01 vs. model group.

The results of α-diversity are shown in Figures 5A,B with normalization in Microbiome Analyst via Total sum scaling (TSS). No significant differences in the α-diversity (both Shannon and Simpson) of fecal microbiota in the model mice after loperamide treatment as compared to normal group were found. However, the administration of *L. rhamnosus* CCFM1068 led to significant increase in α-diversity (Shannon index and Simpison) compared to those of constipation mice. Loperamide treatment also led to significant changes in the β-diversity of the fecal microbiota in the model group relative to the normal group (Figure 5C, *P*_normal vs.model_ = 0.002). According to PCA map of genus level microbiome, the administration of *L. rhamnosus* did not completely restore the structure of the fecal microbiota (*P*_normal vs.CCFM1068_ = 0.002, *P*_normal vs.FHeNJZ7−1_ = 0.02, *P*_normal vs.FTJDG11−1_ = 0.04). However, treatment with these strains led to a partial recovery in the β-diversity and the relief of constipation symptoms (Figure 5C, *P*_FFJND15−L2 vs.model_ = 0.02, *P*_CCFM1068 vs.model_ = 0.03, *P*_HeNJZ7−1 vs.model_ = 0.02, *P*_FTJDG11−1 vs.model_ = 0.02, *P*_FZJHZ11−7 vs.model_ = 0.03). These results suggested that interventions involving different *L. rhamnosus* strains may have different effects on the gut microbiota and the differences in the composition of the microbiota could affect constipation symptoms.

**Figure 6:**
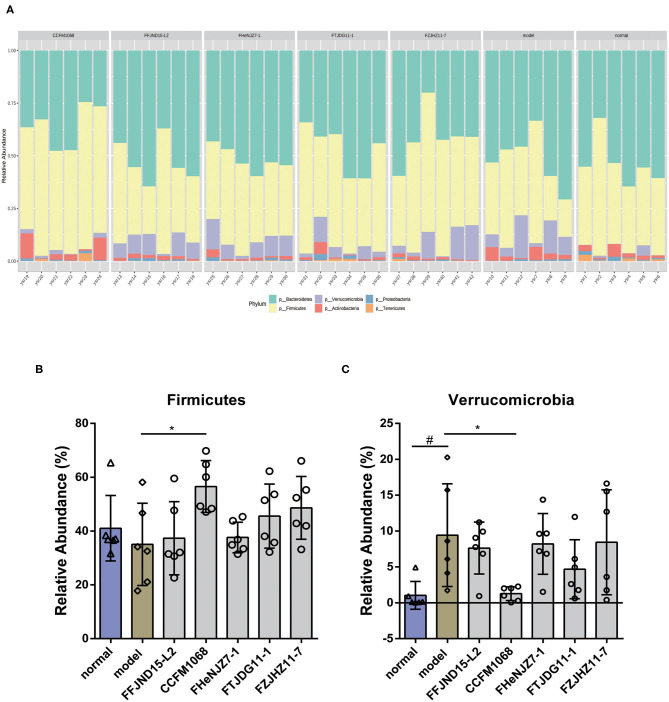
Treatment with *L. rhamnosus* strains alters the phylum-level structure of the gut microbiota. **(A)** Microbial distribution at the phylum level. **(B,C)** Relative abundances of Firmicutes and Verrucomicrobia. Graphs depict mean values ± standard deviations (*n* = 6). Data are means with SEM; Unpaired Student's *t*-test, ^#^*P* < 0.05 vs. normal group; One-way ANOVA followed by Dunnett's multiple comparisons test for *L. rhamnosus*-treated groups, *P < 0.05 vs. model group.

We further investigated phylum-changes in the gut microbiota of mice with the normalization via TSS. The results are shown in Figure 6. Notably, a significant increase of the abundance of Verrucomicrobia in the constipation model mice feces was observed, also a decrease of this phylum abundance in feces after intragastric administration of *L. rhamnosus* CCFM1068 (Figure 6C). These findings suggested that the ability of *L. rhamnosus* CCFM1068 to relieve constipation symptoms may be related to a reduction in the abundance of Verrucomicrobia. In addition, though there were no significant changes in Firmicutes abundance in the model group compared to normal group, *L. rhamnosus* CCFM1068 significantly increased the relative abundance of Firmicutes compared to model group (Figure 6B).

**Figure 7:**
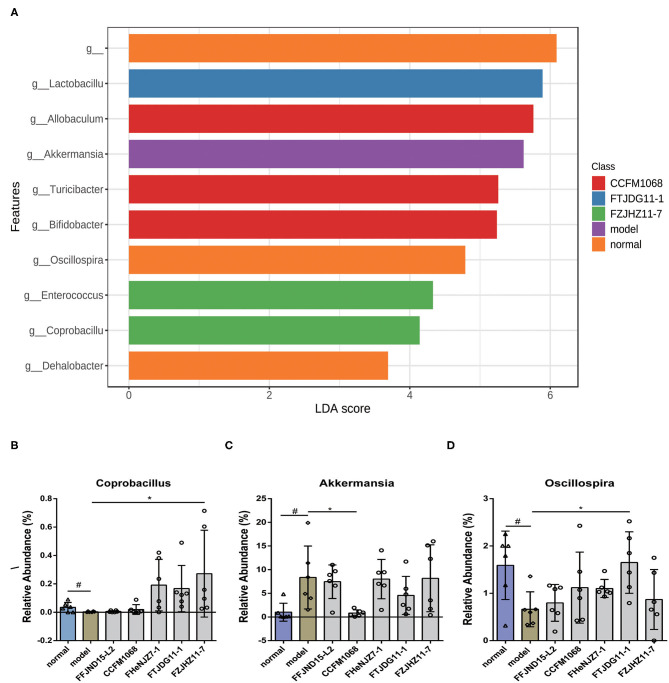
Treatment with *L. rhamnosus* affects the gut microbiota at the genus levels. **(A)** LDA difference analysis. **(B–D)** Relative abundances of the genera *Coprobacillus, Akkermansia* and *Oscillospira*. The graphs depict the mean values ± standard deviations (*n* = 6). Data are means with SEM; Unpaired Student's *t*-test, ^#^*P* < 0.05 vs. normal group; One-way ANOVA followed by Dunnett's multiple comparisons test for *L. rhamnosus*-treated groups, *P < 0.05 vs. model group.

Next, a LEfSe analysis of the fecal microbiota (LDA score 2.0) was conducted to further explore the abilities of the five *L. rhamnosus* strains to alter the microbiota population. Notably, genus-level fecal microbiota profiles varied significantly among the groups of *L. rhamnosus* treated mice. As shown in Figure 7, the abundance of *Coprobacillus* and *Oscillospira* were significantly decreased in the fecal microbiota of the model group relative to the normal group. Whilst treatment of FZJHZ11-7 increased the abundance of *Coprobacillus* in the fecal microbiota relative to the model group. Similar significant increase also occurred on the abundance of *Oscillospira* by FTJDG11-1 treatment (Figures 7B,D). Besides, a significant increase in the abundance of *Akkermansia* was found in the fecal microbiota of the model group relative to the normal group, whilst treatment of CCFM1068 reversed this change in the fecal microbiota of constipated mice (Figure 7C). These results suggest that the different capacities of *L. rhamnosus* strains to relieve constipation may be related to their effects on the abundances of various gut bacteria.

## Discussion

Slow transit constipation is a common syndrome of digestive disorders and is characterized by a low frequency of defecation, dry stools and prolonged bowel movements (Wang et al., 2017b). A prospective study in Japan observed an association of a lower bowel movement frequency with a higher rate of cardiovascular disease-related mortality (Honkura et al., 2016). Clinically, symptoms of constipation are often accompanied by disorders of the gut microbiota. Such disorders can damage the intestinal barrier and induce effects beyond the human digestive system. Probiotics treatment is a recognized method for relieving gut microbiota dysbiosis. Although studies have increasingly explored the abilities of probiotics to alleviate constipation, the specific underlying mechanisms remain unclear. In this study, we investigated the pathways by which *L. rhamnosus* relieves constipation, evaluated potential strain-related differences in these pathways and identify the effects of these strains on the gut microenvironment. Unlike previous studies, *L. rhamnosus* strains used in this study did not relieve constipation by recovering gut SCFAs levels as in the administration of bifidobacteria in earlier studies (Wang et al., 2017a,b, 2019). Besides, different strains of *L. rhamnosus* also showed differences in the way to relieve constipation.

Shi et al. reported significantly reduced fecal levels of SCFfacetic acid, propionic acid, and butyric acid) with constipation than in the normal population (Shi et al., 2016). These observations indicate a certain relationship between the occurrence of constipation and the intestinal levels of SCFAs. So SCFAs produced by intestinal flora or probiotics were believed to be effective on constipation alleviation. Moreover, it was reported that the administration of *Lactobacillus plantarum* NCU116 significantly improved the symptoms of constipation in mice and led to significant increases of acetic acid and propionic acid levels in their feces (Li et al., 2015). Although lactic acid bacteria relieve constipation and simultaneously induce changes in SCFAs levels, the roles of these SCFAs in the alleviation of constipation remain unclear. Unexpectedly, in this study, the five strains of *L. rhamnosus* failed to recover the fecal levels of SCFAs, suggesting that different mechanisms could mediate the relief of constipation symptoms associated with these species of probiotics.

The existing literature indicates that an increase in systemic PYY levels can slow GI emptying (Hagan, 2002; Parnell and Reimer, 2009). Furthermore, an increase in the number of colonic PYY cells and the synthesis of PYY have been identified as major causes of slow transit constipation (El-Salhy et al., 2013). The results of this study indicated that the administration of loperamide to induce constipation led to significant increases in the serum PYY levels of the mice. Other studies have shown that the gut microbiota affects systemic PYY levels (Duca et al., 2012). Taken together, our and previous results lead us to believe that constipation could be alleviated by a probiotic intervention through decreasing the PYY levels in serum. Psichas et al. postulated that PYY secretion is mediated via SCFAs receptors expressed on the surfaces of L cells (Psichas et al., 2015). However, our observation that treatment with *L. rhamnosus* did not significantly enhance fecal SCFAs levels suggests that PYY secretion may be mediated via other mechanisms.

Patients with constipation often present with abnormal enteric nervous system functions, and previous reports on constipation have described reduced numbers of colonic neurons and damage to this cell population (Bassotti et al., 2007; Chandrasekharan et al., 2011). NT-3 and BDNF play key roles in the development and maturation of neurons and promote the regeneration of intestinal neurons (Sterne et al., 1997; Hansebout et al., 2012). Moreover, increased levels of exogenous NT-3 and BDNF can accelerate colonic peristalsis and increase the frequency of defecation (Parkman et al., 2003; Chen et al., 2014). In this study, the increased NT-3 and BDNF levels induced by individual *L. rhamnosus* strains suggest that some probiotic strains may specifically promote the production of NT-3 and BDNF in the colon and thereby relieve constipation.

Evidence from rat study suggests that SCFAs can directly increase the level of 5-HT in the colonic lumen *in vitro* and thus improve colonic transmission (Fukumoto et al., 2003). *In vivo* experiments further revealed the abilities of intestinal microbiota to stimulate 5-HT secretion from intestinal chromaffin cells by increasing the levels of SCFAs in the colon, thereby promoting intestinal transmission (Reigstad et al., 2015). Previous study also suggested that the metabolites of certain spore-forming anaerobic bacteria in the gut microbiota can affect the level of 5-HT and thereby affect intestinal motility (Yano et al., 2015). It was also reported in the literature that *Bacteroides* spp. accelerated intestinal peristalsis by modulating the synthesis of tryptophan, an important precursor for the synthesis of 5-HT, in germ-free mice (Bhattarai et al., 2018). Surprisingly, in this study several specific *L. rhamnosus* strains increased the level of 5-HT in the colon without significantly affecting the intestinal levels of SCFAs. These findings lead us to speculate that *L. rhamnosus* may increase the level of 5-HT through an as-yet unknown pathway, in addition to the SCFAs-mediated pathway.

Several clinical population and animal experiments have demonstrated an inextricable link between the symptoms of constipation and the gut microbiota. Ge et al. observed an elevated abundance of Verrucomicrobia in the feces of patients with slow-transit constipation, consistent with the findings from this study (Ge et al., 2017). Furthermore, a species analysis in this study revealed that treatment with *L. rhamnosus* CCFM1068 restored the relative abundance of Verrucomicrobia in constipated mice to normal levels. We speculate that recovering the abundance of Verrucomicrobia may be an important way for CCFM 1068 to alleviate constipation. The genus analysis in this study also revealed that mice with constipation exhibited a significant decrease in the relative abundance of *Oscillospira* when compared to mice in the normal group, consistent with the findings of a previous study (Ge et al., 2017). Previous research has revealed a strong association between *Oscillospira* and obesity (Beaumont et al., 2016). Also *Oscillospira* spp. was reported to show less abundant in obese humans than those in leaner counterparts (Konikoff and Gophna, 2016). In this study, the association between constipation and *Oscillospira* was also observed. Considering the low levels of inflammation exists both in constipated mice and human (Mokhtare et al., 2017; Ren et al., 2017), and the reduced abundance of *Oscillospira* in human gut with inflammation (Walters et al., 2014), it is speculated that the change of *Oscillospira* may be closely related to the development of constipation.

Increased in the abundance of *Akkermansia* in feces of constipated mice was observed in this study. *Akkermansia muciniphila* is widely accepted as the new generation of probiotics because of its potential anti-inflammatory properties (Derrien et al., 2017). However, increasing abundance of *Akkermansia* was also observed in the patients suffering from colon cancer, indicating a complex relationship between *Akkermansia* and diseases (Hibberd et al., 2017). So whether *Akkermansia* participates in constipation development still need to be confirmed.

## Conclusions

Increasing studies have confirmed that slow-transit constipation is closely related to gut microbiota dysbiosis. However, the etiology of slow transit constipation remains unclear. Our previous study found that Bifidobacteria relieve constipation is associated with recovery of SCFAs levels. However, in this study, we observed that the abilities of *L. rhamnosus* strains to improve constipation symptoms were not associated with increased levels of SCFAs in the colon. Different strains of *L. rhamnosus* had various effects on different aspects of the GI tract in constipation model mice, including the secretion of motility-regulating peptides, neurotransmitters and neurotrophic factors and the composition of the gut microbiota. The findings suggest that different strains of the same lactic acid bacterial species may improve constipation via different mechanisms associated with inter-strain differences in the cell surface molecules and metabolites. Therefore, combinations of strains with different efficiencies may yield superior relief from constipation symptoms. These observations could be useful for future screening of probiotics for the relief of constipation symptoms. However, this study still needs to be improved due to the lack of control experiments with naloxone or other opioid antagonist that would have indicated the optimum anti-constipating action. Comparing with some standard medicines for constipation will help for investigating whether the probiotics can be used as a dietary strategy for constipation alleviation or even a substitute for medicine. Also, investigation of microbiota in different intestinal segments will help to identify more biomarker of gut microbe for constipation.

## Data Availability Statement

The datasets generated for this study can be found in the NCBI, BioProject: PRJNA578847, Taxonomy ID: 1861841, https://www.ncbi.nlm.nih.gov/bioproject/PRJNA578847.

## Ethics Statement

The animal study was reviewed and approved by The Ethics Committee of Jiangnan University (JN.No20180115b1920520).

## Author Contributions

GWa, GWu, and WC conceived and designed the experiments. GWa, SY, SS, QS, and LW performed the experiments. GWa and SY analyzed the data. GWu, QZ, JZ, HZ, and WC contributed reagents, materials, and analysis tools. GWa and SY wrote the paper. All authors contributed to manuscript revision, read and approved the submitted version.

## Conflict of Interest

The authors declare that the research was conducted in the absence of any commercial or financial relationships that could be construed as a potential conflict of interest.

## References

[B1] Aoki R. Tsuchida S. Arai Y. Ohno K. Nishijima T. Mawatari T.. (2016). Effect of *Bifidobacterium animalis* subsp. lactis GCL2505 on the physiological function of intestine in a rat model. Food Sci. Nutr. 4, 782–790. 10.1002/fsn3.344 27826427 PMC5090641

[B2] Bassotti G. Villanacci V. Fisogni S. Cadei M. Galletti A. Morelli A.. (2007). Comparison of three methods to assess enteric neuronal apoptosis in patients with slow transit constipation. Apoptosis 12, 329–332. 10.1007/s10495-006-0572-0 17191125

[B3] Beaumont M. Goodrich J. K. Jackson M. A. Yet I. Davenport E. R. Vieira-Silva S.. (2016). Heritable components of the human fecal microbiome are associated with visceral fat. Genome Biol. 17:189. 10.1186/s13059-016-1052-7 27666579 PMC5036307

[B4] Bhattarai Y. Williams B. B. Battaglioli E. J. Whitaker W. R. Till L. Grover M.. (2018). Gut microbiota-produced tryptamine activates an epithelial g-protein-coupled receptor to increase colonic secretion. Cell Host Microbe 23, 775–785.e5. 10.1016/j.chom.2018.05.004 29902441 PMC6055526

[B5] Chandrasekharan B. Anitha M. Blatt R. Shahnavaz N. Kooby D. Staley C.. (2011). Colonic motor dysfunction in human diabetes is associated with enteric neuronal loss and increased oxidative stress. Neurogastroenterol. Motil. 23, 131–138.e26. 10.1111/j.1365-2982.2010.01611.x 20939847 PMC3020997

[B6] Chen F. Yu Y. Wang P. Dong Y. Wang T. Zuo X.. (2014). Brain-derived neurotrophic factor accelerates gut motility in slow-transit constipation. Acta Physiol. 212, 226–238. 10.1111/apha.12374 25164090

[B7] Choopani R. Ghourchian A. Hajimehdipoor H. Kamalinejad M. Ghourchian F. (2017). Effect of *Descurainia sophia* (L.) webb ex prantl on adult functional constipation: a prospective pilot study. J. Evidence Based Complementary Altern. Med. 22, 1–6. 10.1177/2156587217703018 28401774 PMC5871276

[B8] Derrien M. Belzer C. De Vos W. M. (2017). *Akkermansia muciniphila* and its role in regulating host functions. Microb. Pathog. 106, 171–181. 10.1016/j.micpath.2016.02.005 26875998

[B9] Dhariwal A. Chong J. Habib S. King I. L. Agellon L. B. Xia J. (2017). Microbiomeanalyst: a web-based tool for comprehensive statistical, visual and meta-analysis of microbiome data. Nucleic Acids Res. 45, W180–W188. 10.1093/nar/gkx295 28449106 PMC5570177

[B10] Dimidi E. Christodoulides S. Scott S. M. (2017). Mechanisms of action of probiotics and the gastrointestinal microbiota on gut motility and constipation. Adv. Nutr. 8, 484–494. 10.3945/an.116.014407 28507013 PMC5421123

[B11] Duca F. A. Swartz T. D. Sakar Y. Covasa M. (2012). Increased oral detection, but decreased intestinal signaling for fats in mice lacking gut microbiota. PLoS ONE 7:e39748. 10.1371/journal.pone.0039748 22768116 PMC3387243

[B12] El-Salhy M. Mazzawi T. Gundersen D. Hatlebakk J. G. Hausken T. (2013). The role of peptide YY in gastrointestinal diseases and disorders (review). Int. J. Mol. Med. 31, 275–282. 10.3892/ijmm.2012.1222 23292145 PMC4042877

[B13] Fukumoto S. Tatewaki M. Yamada T. Fujimiya M. Mantyh C. Voss M.. (2003). Short-chain fatty acids stimulate colonic transit via intraluminal 5-HT release in rats. Am. J. Physiol. Regul. Integr. Comp. Physiol. 284, R1269–R1276. 10.1152/ajpregu.00442.2002 12676748

[B14] Ge X. Zhao W. Ding C. Tian H. Xu L. Wang H.. (2017). Potential role of fecal microbiota from patients with slow transit constipation in the regulation of gastrointestinal motility. Sci. Rep. 7:441. 10.1038/s41598-017-00612-y 28348415 PMC5428802

[B15] Hagan M. M. (2002). Peptide YY: a key mediator of orexigenic behavior. Peptides 23, 377–382. 10.1016/S0196-9781(01)00614-3 11825652

[B16] Hansebout C. R. Su C. Reddy K. Zhang D. Jiang C. Rathbone M. P.. (2012). Enteric glia mediate neuronal outgrowth through release of neurotrophic factors. Neural Regen. Res. 7, 2165–2175. 10.3969/j.issn.1673-5374.2012.028.001 25538736 PMC4268714

[B17] Hibberd A. A. Lyra A. Ouwehand A. C. Rolny P. Lindegren H. Cedgrd L.. (2017). Intestinal microbiota is altered in patients with colon cancer and modified by probiotic intervention. BMJ Open Gastroenterol. 4:e000145. 10.1136/bmjgast-2017-000145 28944067 PMC5609083

[B18] Honkura K. Tomata Y. Sugiyama K. Kaiho Y. Watanabe T. Zhang S.. (2016). Defecation frequency and cardiovascular disease mortality in Japan: the ohsaki cohort study. Atherosclerosis 246, 251–256. 10.1016/j.atherosclerosis.2016.01.007 26812003

[B19] Kim S. E. Choi S. C. Park K. S. Park M. I. Shin J. E. Lee T. H.. (2015). Change of fecal flora and effectiveness of the short-term VSL#3 probiotic treatment in patients with functional constipation. J. Neurogastroenterol. Motil. 21, 111–120. 10.5056/jnm14048 25537674 PMC4288088

[B20] Koliani J. Lacy B. E. (2017). Update on the management of chronic constipation. Curr. Treat. Options Gastroenterol. 15, 126–134. 10.1007/s11938-017-0118-2 28116695

[B21] Konikoff T. Gophna U. (2016). *Oscillospira*: a central, enigmatic component of the human gut microbiota. Trends Microbiol. 24, 523–524. 10.1016/j.tim.2016.02.015 26996766

[B22] Li C. Nie S. P. Zhu K. X. Xiong T. Li C. Gong J.. (2015). Effect of *Lactobacillus plantarum* NCU116 on loperamide-induced constipation in mice. Int. J. Food Sci. Nutr. 66, 533–538. 10.3109/09637486.2015.1024204 25822005

[B23] Matsumoto K. Takada T. Shimizu K. Moriyama K. Kawakami K. Hirano K.. (2010). Effects of a probiotic fermented milk beverage containing *Lactobacillus casei* strain shirota on defecation frequency, intestinal microbiota, and the intestinal environment of healthy individuals with soft stools. J. Biosci. Bioeng. 110, 547–552. 10.1016/j.jbiosc.2010.05.016 20580604

[B24] Mokhtare M. Alimoradzadeh R. Agah S. Mirmiranpour H. Khodabandehloo N. (2017). The association between modulating inflammatory cytokines and constipation of geriatrics in Iran. Middle East J. Dig. Dis. 9, 228–234. 10.15171/mejdd.2017.78 29255581 PMC5726336

[B25] Parkman H. P. Rao S. S. C. Reynolds J. C. (2003). Neurotrophin-3 improves functional constipation. Am. J. Gastroenterol. 98, 1338–1347. 10.1111/j.1572-0241.2003.t01-1-07477.x 12818279

[B26] Parnell J. A. Reimer R. A. (2009). Weight loss during oligofructose supplementation is associated with decreased ghrelin and increased peptide YY in overweight and obese adults. Am. J. Clin. Nutr. 89, 1751–1759. 10.3945/ajcn.2009.27465 19386741 PMC3827013

[B27] Psichas A. Sleeth M. L. Murphy K. G. Brooks L. Bewick G. A. Hanyaloglu A. C.. (2015). The short chain fatty acid propionate stimulates GLP-1 and PYY secretion via free fatty acid receptor 2 in rodents. Int. J. Obes. 39, 424–429. 10.1038/ijo.2014.153 25109781 PMC4356745

[B28] Qian Y. Suo H. Du M. Zhao X. Li J. Li G. J.. (2015). Preventive effect of *Lactobacillus fermentum* Lee on activated carbon-induced constipation in mice. Exp. Ther. Med. 9, 272–278. 10.3892/etm.2014.2064 25452815 PMC4247307

[B29] Reigstad C. S. Salmonson C. E. Rainey J. F. III. Szurszewski J. H. Linden D. R. Sonnenburg J. L.. (2015). Gut microbes promote colonic serotonin production through an effect of short-chain fatty acids on enterochromaffin cells. FASEB J. 29, 1395–1403. 10.1096/fj.14-259598 25550456 PMC4396604

[B30] Ren X. Liu L. Gamallat Y. Zhang B. Xin Y. (2017). Enteromorpha and polysaccharides from enteromorpha ameliorate loperamide-induced constipation in mice. Biomed. Pharmacother. 96, 1075–1081. 10.1016/j.biopha.2017.11.119 29198923

[B31] Sharma A. Rao S. (2016). Constipation: pathophysiology and current therapeutic approaches. Handb. Exp. Pharmacol. 239, 59–74. 10.1007/164_2016_111 28185025

[B32] Shi Y. Chen Q. Huang Y. (2016). Function and clinical implications of short-chain fatty acids in patients with mixed refractory constipation. Colorectal Dis. 18, 803–810. 10.1111/codi.13314 26921846

[B33] Sterne G. D. Brown R. A. Green C. J. (1997). Neurotrophin-3 delivered locally via fibronectin mats enhances peripheral nerve regeneration. Eur. J. Neurosci. 9, 1388–1396. 10.1111/j.1460-9568.1997.tb01493.x 9240396

[B34] Walters W. A. Xu Z. Knight R. (2014). Meta-analyses of human gut microbes associated with obesity and IBD. FEBS Lett. 588, 4223–4233. 10.1016/j.febslet.2014.09.039 25307765 PMC5050012

[B35] Wang L. Chen C. Cui S. Lee Y. K. Wang G. Zhao J.. (2019). Adhesive bifidobacterium induced changes in cecal microbiome alleviated constipation in mice. Front. Microbiol. 10:1721. 10.3389/fmicb.2019.01721 31456752 PMC6700325

[B36] Wang L. Hu L. Xu Q. Jiang T. Fang S. Wang G.. (2017a). Bifidobacteria exert species-specific effects on constipation in BALB/c mice. Food Funct. 8, 3587–3600. 10.1039/C6FO01641C 28884754

[B37] Wang L. Hu L. Xu Q. Yin B. Fang D. Wang G.. (2017b). Bifidobacterium adolescentis exerts strain-specific effects on constipation induced by loperamide in BALB/c Mice. Int. J. Mol. Sci. 18:318. 10.3390/ijms18020318 28230723 PMC5343854

[B38] Yano J. M. Yu K. Donaldson G. P. Shastri G. G. Ann P. Ma L.. (2015). Indigenous bacteria from the gut microbiota regulate host serotonin biosynthesis. Cell 161, 264–276. 10.1016/j.cell.2015.02.047 25860609 PMC4393509

[B39] Zhao Y. Yu Y. B. (2016). Intestinal microbiota and chronic constipation. Springerplus 5:1130. 10.1186/s40064-016-2821-1 27478747 PMC4951383

[B40] Zoppi G. Cinquetti M. Luciano A. (1998). The intestinal ecosystem in chronic functional constipation. Acta Paediatr. 87, 836–841. 10.1080/080352598750013590 9736230

