# Supplementary material for: Lactobacillus rhamnosus Strains Relieve Loperamide-Induced Constipation via Different Pathways Independent of Short-Chain Fatty Acids
Source: Front Cell Infect Microbiol. 2020 Aug 19;10:423. doi: 10.3389/fcimb.2020.00423 (PMC7466723; doi:10.3389/fcimb.2020.00423)
Supplement: Supplementary file 1 [file Data_Sheet_1.pdf]

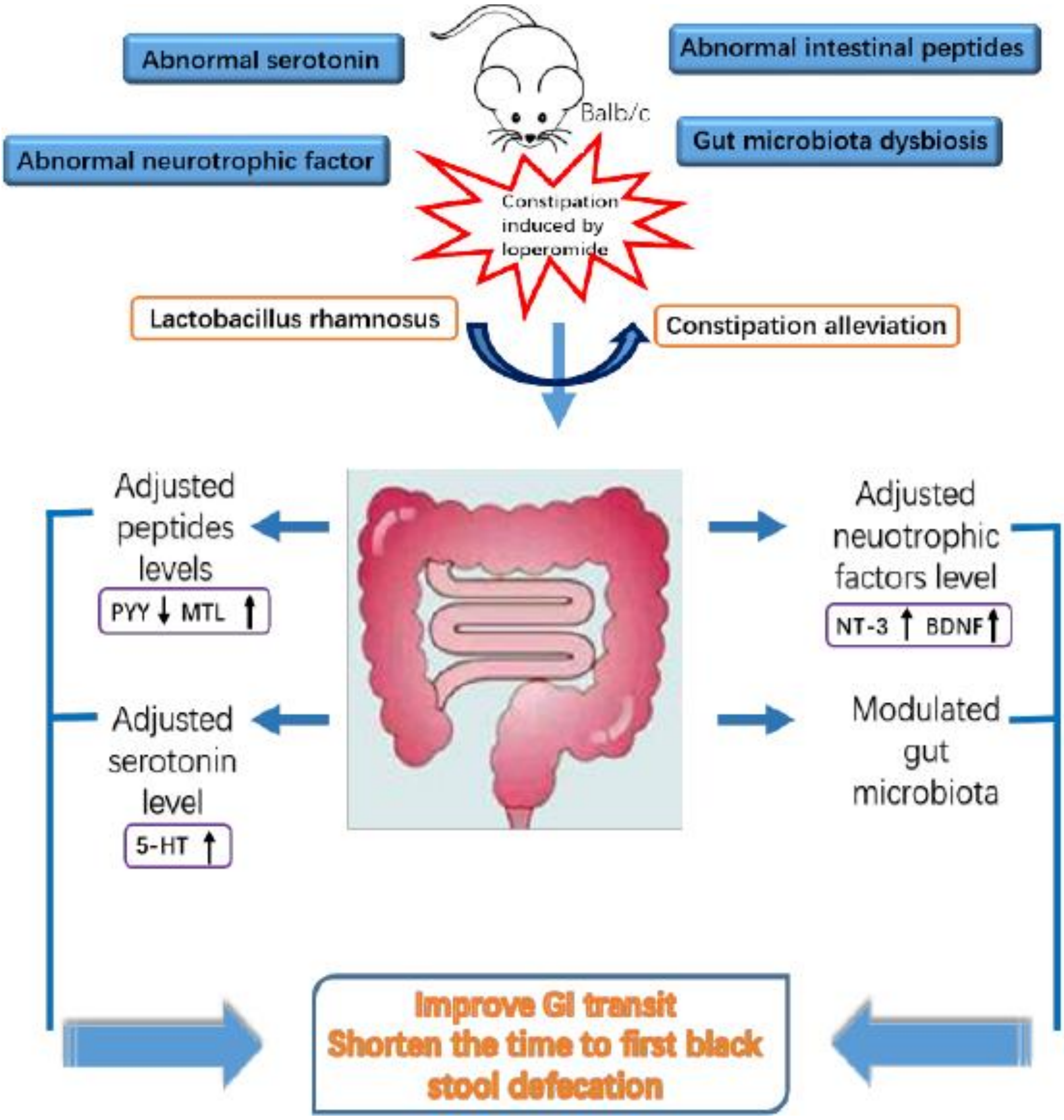

Lactobacillus rhamnosus Strains Relieve Loperamide-Induced Constipation via Different Pathways Independent of Short-Chain Fatty Acids
